# Effect of Myostatin Depletion on Weight Gain, Hyperglycemia, and Hepatic Steatosis during Five Months of High-Fat Feeding in Mice

**DOI:** 10.1371/journal.pone.0017090

**Published:** 2011-02-24

**Authors:** Kerri Burgess, Tianshun Xu, Roger Brown, Bajin Han, Stephen Welle

**Affiliations:** 1 Endocrinology and Metabolism Division, Department of Medicine, University of Rochester, Rochester, New York, United States of America; 2 Muscle Metabolism DPU and Platform Technology and Science, GlaxoSmithKline Research and Development, Research Triangle Park, North Carolina, United States of America; University of California, Los Angeles, and Cedars-Sinai Medical Center, United States of America

## Abstract

The marked hypermuscularity in mice with constitutive myostatin deficiency reduces fat accumulation and hyperglycemia induced by high-fat feeding, but it is unclear whether the smaller increase in muscle mass caused by postdevelopmental loss of myostatin activity has beneficial metabolic effects during high-fat feeding. We therefore examined how postdevelopmental myostatin knockout influenced effects of high-fat feeding. Male mice with ubiquitous expression of tamoxifen-inducible Cre recombinase were fed tamoxifen for 2 weeks at 4 months of age. This depleted myostatin in mice with floxed myostatin genes, but not in control mice with normal myostatin genes. Some mice were fed a high-fat diet (60% of energy) for 22 weeks, starting 2 weeks after cessation of tamoxifen feeding. Myostatin depletion increased skeletal muscle mass ∼30%. Hypermuscular mice had ∼50% less weight gain than control mice over the first 8 weeks of high-fat feeding. During the subsequent 3 months of high-fat feeding, additional weight gain was similar in control and myostatin-deficient mice. After 5 months of high-fat feeding, the mass of epididymal and retroperitoneal fat pads was similar in control and myostatin-deficient mice even though myostatin depletion reduced the weight gain attributable to the high-fat diet (mean weight with high-fat diet minus mean weight with low-fat diet: 19.9 g in control mice, 14.1 g in myostatin-deficient mice). Myostatin depletion did not alter fasting blood glucose levels after 3 or 5 months of high-fat feeding, but reduced glucose levels measured 90 min after intraperitoneal glucose injection. Myostatin depletion also attenuated hepatic steatosis and accumulation of fat in muscle tissue. We conclude that blocking myostatin signaling after maturity can attenuate some of the adverse effects of a high-fat diet.

## Introduction

When laboratory mice are fed a high-fat diet, they become obese and have hyperglycemia and hepatic steatosis as often observed in obese humans. This diet-induced obesity is attenuated in mice in which there is marked hypermuscularity caused by constitutive absence of functional myostatin or constitutive expression of a protein that inhibits myostatin activity [1]–[5]. Even on a low-fat diet, mice with constitutive myostatin deficiency are leaner than normal mice [3], [4], [6]–[8]. Mice with obesity-inducing genetic mutations also are leaner when myostatin is absent [6]. Because muscle is a significant glucose consumer when insulin levels are high, and because leanness improves sensitivity to insulin, it is not surprising that constitutive myostatin deficiency reduces hyperglycemia after a glucose challenge in mice with obesity-inducing mutations [6] and in mice fed a high-fat diet [1], [3], [4]. A constitutive myostatin gene mutation attenuated the hepatic steatosis induced by a high-fat diet [4].

The studies cited above raise the possibility that an anti-myostatin therapy, if one can be developed, would reduce fat mass, glucose levels, and hepatic steatosis in obese humans. However, at least in mice, the increase in muscle mass that can be achieved by blocking myostatin activity after muscle development is only about one-fourth to one-third of the increase in muscle mass caused by constitutive myostatin deficiency [9]–[12]. In old mice, systemic administration of an anti-myostatin antibody for four weeks did not affect fat mass or glucose levels, but muscle mass increased only ∼15% and the mice were not challenged with a high-fat diet [13]. In very young *ob/ob* mice, administration of an anti-myostatin antibody for 6 weeks increased muscle mass by about one-third but did not attenuate the rapid increase in adiposity during this period [14]. Nevertheless, the myostatin antibody reduced blood glucose levels. In normal young mice, systemic administration of a soluble activin receptor type IIB (RAP-031, which blocks the activity of myostatin and other ligands that bind to this receptor) increased muscle mass by about one-third and markedly reduced the increase in fat mass that occurred between 4 and 10 weeks of high-fat feeding [9]. Serum glucose levels after 10 weeks of high-fat feeding also were reduced by RAP-031 administration. Because myostatin was inhibited by a nonspecific mechanism in that study, it is unclear to what extent the reduction of myostatin activity was responsible for these effects. In the present study, we used a genetic approach to specifically eliminate myostatin activity after normal development, and we examined how this affected weight gain, muscle mass, fat mass, glucose levels and hepatic steatosis after a prolonged period of high-fat feeding.

## Methods

All procedures were approved by the University of Rochester animal research committee and were consistent with all regulations on humane use of animals for research (PHS Assurance Number A–3292–01, Protocol Number 100624/2007–029).

Mice in which exon 3 of the myostatin gene is flanked by loxP sequences (Mstn[f/f]) have been described previously [11], [15]. These mice, and Mstn[w/w] controls (normal myostatin genes), were bred with mice hemizygous for the CAGG-CreER transgene [16]. Cre+male mice (both Mstn[f/f] and Mstn[w/w]) were used in this research. Genotyping was carried out as described previously [11]. The background strain for all mice was C57BL/6. Mice received food and water ad libitum and were kept in MicroVENT cages (Allentown Caging), 2–3 mice per cage, in a room with controlled lighting (12 light cycle starting 0600 h) and temperature (74°C).

When the mice were 4 months old, their usual low-fat chow (LabDiet 5010, 12.7% of energy from fat, 58.5% from carbohydrates, 28.7% from protein) was withdrawn and replaced with low-fat chow containing tamoxifen (250 mg/kg) for a two week period to activate the CreER protein. This procedure knocks out myostatin exon 3 in Mstn[f/f]/CreER+mice [11], [17]. Loss of myostatin was confirmed by immunoblotting [18]. Mstn[w/w] control mice also received tamoxifen chow to control for any nonspecific effects of the drug or the presence of activated CreER. After tamoxifen-containing chow was withdrawn, all mice were fed LabDiet 5010 for two weeks.

Two weeks after the completion of tamoxifen administration, some of the mice were switched to a high-fat diet (Research Diets D12462, 60% of energy from fat, 20% from carbohydrates, 20% from protein). Other mice continued to consume the low-fat LabDiet 5010. The mice stayed on the assigned diets for 22 weeks. They were weighed at 2 week intervals. Mandibular vein glucose concentrations were determined with a FreeStyle Freedom™glucose meter (Abbott) during the 13^th^ and 21^st^ week of high-fat feeding. Blood samples were obtained in the afternoon, 5 hr after chow was removed from the cage, both before and after ip injection of glucose (1 mg/g body weight). Initially, we tested glucose concentrations at 30, 60, and 90 min after glucose injection. This frequent sampling protocol was stressful to the mice and resulted in highly variable and sometimes unexpectedly high glucose concentrations even in mice consuming normal chow. Hence, we changed the protocol to sample blood only once after the glucose injection, at 90 min. Mean fasting glucose levels reported here include data from all mice, but the mean 90 min post-injection levels are based on only those mice that had a single post-injection blood sample.

After 22 weeks of high-fat feeding, or the same period of low-fat feeding, mice were euthanized for determination of the mass of skeletal muscles (gastrocnemius, quadriceps, triceps), heart, liver, kidney, and intra-abdominal fat pads (retroperitoneal and epididymal, bilateral). In mice fed the low-fat diet, retroperitoneal and epididymal fat pads accounted for nearly all of the visible adipose tissue within the abdominal cavity, except for very small amounts adhering to intestine and pancreas. In mice fed the high-fat diet, adipose tissue surrounding gastrointestinal organs appeared to be continuous with the retroperitoneal adipose tissue and was classified as such. Muscles were snap frozen in liquid nitrogen and stored at −70°C. Livers were stored in 10% buffered neutral formalin. Fatty acid contents of triceps muscles, which included lipids within muscle fibers and in adipose tissue between fascicles, were determined by gas chromatography with C17∶0 as an internal standard [19]. Transverse sections of the liver, 2–3 mm thick, were taken from the left lateral lobe and were transferred to an osmium tetroxide-potassium dichromate solution for 8 hours. The tissues were rinsed in running tap water for 2 hours and processed overnight on a tissue processor. After embedding in paraffin, 5 micron sections were cut and stained with Hematoxylin and Eosin. Sections were graded for hepatocellular steatosis according to the following 4-point scale: 1) rare individual hepatocytes containing intermediate to microvesicular lipid droplets; 2) midzonal to centrilobular distribution of primarily small to intermediate macro-vesicular and micro-vesicular lipid droplets with less than 50% of the lobules affected; 3) midzonal to centrilobular distribution of intermediate-size macrovesicular and microvesicular lipid droplets, smaller in size than grade 4 and involving less than 70% of the lobules; 4) midzonal to centrilobular distribution of numerous large macrovesicular and microvesicular lipid droplets distributed across more than 70% of the lobules.

Data are expressed as means, and where error terms are shown these are standard errors of the mean. Factorial analysis of variance (ANOVA; myostatin status×time, with time as a within-subject factor) was done to assess the statistical significance of the effect of myostatin depletion on biweekly weight changes and biweekly cumulative weight gain. The statistical significance of the effect of myostatin depletion at each time point was assessed by Bonferroni t-tests to adjust *P* for multiple comparisons. The denominator for computing the t values was based on the residual variance from the ANOVA. We analyzed the high-fat and low-fat conditions separately rather than adding dietary condition as an additional factor in the ANOVA, because variance was greater for the high-fat condition. Thus, using residual errors pooled across dietary conditions would have inflated the statistical significance of the effects of myostatin deficiency during high-fat feeding. Variance was higher with the high-fat diet for several other outcome measures, including fat pad mass, liver mass, glucose levels after ip glucose injection, and intramuscular lipid content. We therefore assessed the effects of myostatin depletion separately for the low-fat and high-fat conditions rather than performing ANOVA with diet as a factor. *P* values for effects of myostatin depletion within diet groups were determined with t-tests. Two-tailed *P* values are reported. For simplicity and consistency, this approach was taken for all outcome measures even when variance was similar in low-fat and high-fat conditions. The statistical significance of differences in outcome measures for high-fat feeding versus low-fat feeding in both myostatin-deficient and control mice were determined by t-tests adjusted for unequal variance between groups. Fisher's exact probability test (two-sided) was used to determine whether the distribution of steatosis scores in mice fed the high-fat diet was significantly different in control and myostatin-deficient mice. R version 2.12.0 was used for ANOVA and Fisher's test, and Microsoft Excel was used for t-tests.

## Results

Before tamoxifen administration, the mean body mass of Mstn[f/f] mice was slightly greater than that of Mstn[w/w] mice, 27.6 vs. 26.1 g (*P*<0.01). Mice of both genotypes lost some body mass during 2 weeks of tamoxifen feeding (mean 2.2 g in Mstn[f/f] and 1.8 g in Mstn[w/w], *P*>0.3 for genotype difference). During the first 2 weeks after tamoxifen feeding, before the mice started consuming the high-fat diet, Mstn[f/f] mice gained more weight than Mstn[w/w] mice (4.3 vs. 2.5 g, *P*<0.001), presumably because of a rapid increase in muscle mass (in a recent unpublished study, we determined that gastrocnemius and quadriceps muscles were 25–30% larger in Mstn[f/f]/CreER+ mice than in Mstn[w/w]/CreER+ mice 2 weeks after the cessation of tamoxifen feeding). Thus, body mass was greater (*P*<0.001) in myostatin-depleted mice at the onset of high-fat feeding (29.1 vs. 26.5 g) and at the same time point in mice that continued on the low-fat diet (30.1 vs. 27.0 g). After the more rapid weight gain in Mstn[f/f] mice during the first 2 weeks post-tamoxifen, there was no further effect of myostatin depletion on the increment in total body mass in mice in which the low-fat diet was continued during the final 22 weeks of the study (3.8 g in myostatin-deficient mice; 3.6 g in normal mice).

**Figure 1:**
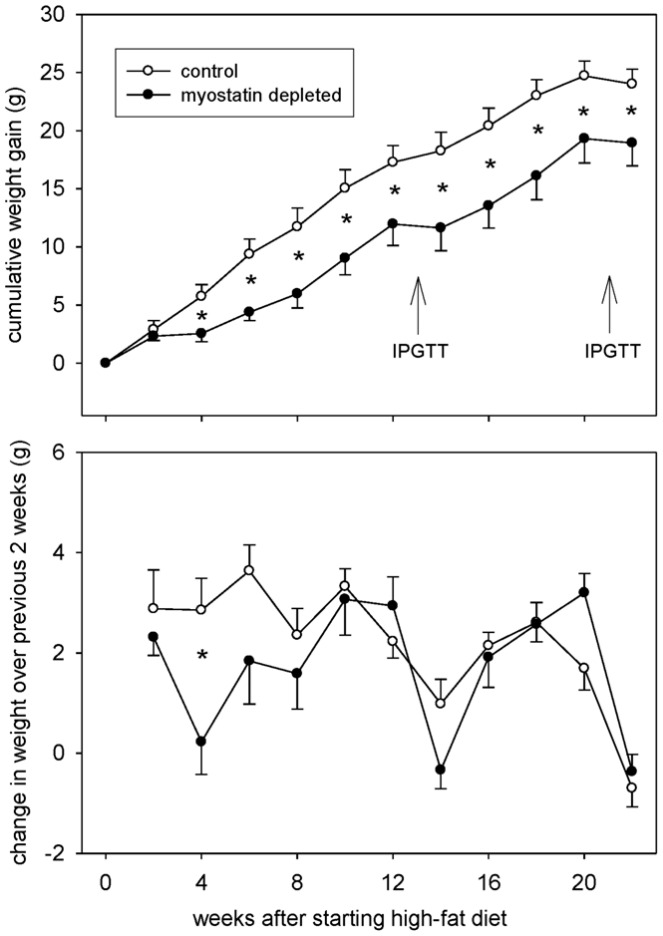
Mean (±SEM) change in total body mass. Top panel shows cumulative weight gains after changing dietary fat from 13% to 60% of energy, and lower panel shows biweekly weight changes independent of previous measures. IPGTT = intraperitoneal glucose tolerance test. *P<0.05 (adjusted for multiple comparisons) for difference between normal and myostatin-depleted.

Over the first 8 weeks of high-fat feeding, the cumulative mean weight gain of the myostatin-deficient mice was only half that of the mice with normal myostatin expression (Figure 1, upper panel). Beyond 8 weeks, the rate of weight gain was similar in myostatin-deficient and control mice, but cumulative weight gain remained lower in the myostatin-deficient mice. By ANOVA, the pattern of biweekly weight changes (Figure 1, lower panel) was significantly different in control and myostatin-deficient mice (*P*<0.05 for myostatin status×time interaction). The only individual time interval with significantly lower weight gain (*P*<0.05) in myostatin-deficient mice was the interval between 2 and 4 weeks after starting the high-fat diet. Myostatin-deficient mice had a greater body mass when the high-fat diet was started (because of increased muscle mass), so their smaller weight gains did not result in significantly lower body mass at any time point (*P*>0.1, not shown except for final weights in Table 1).

**Table 1:** Effects of myostatin depletion and high-fat feeding on body and organ mass.

	Low-fat diet		High-fat diet	
	Normal myostatin	Myostatin depleted	Normal myostatin	Myostatin depleted
Body (g)	30.6±0.5	33.9±0.6^a^	50.5±1.2^b^	48.0±2.0^b^
Epididymal fat (g)	0.58±0.07	0.52±0.06	2.32±0.14^b^	2.68±0.17^b^
Retroperitoneal fat (g)	0.30±0.06	0.29±0.04	2.47±0.16^b^	1.94±0.25^b^
Liver (g)	1.65±0.05	1.64±0.04	2.14±0.14^b^	1.73±0.09^a^
Kidney (mg)	193±5	169±4^a^	192±5	182±7
Heart muscle (mg)	130±3	129±3	149±4^b^	142±3^b^
Gastrocnemius muscles (mg)	146±4	196±5^a^	159±4^b^	200±2^a^
Quadriceps muscles (mg)	223±4	281±7^a^	210±6	277±6^a^
Triceps muscles (mg)	128±2	178±6^a^	126±2	183±7^a^

Values are mean±standard error. Organ mass was determined when mice were 10 months old. Myostatin depletion was induced by tamoxifen feeding for two weeks when mice were 4 months old. High-fat feeding started when mice were 5 months old.

a *P*<0.02 versus normal myostatin group on same diet.

b *P*<0.03 versus low-fat diet group with same myostatin status.

**Figure 2:**
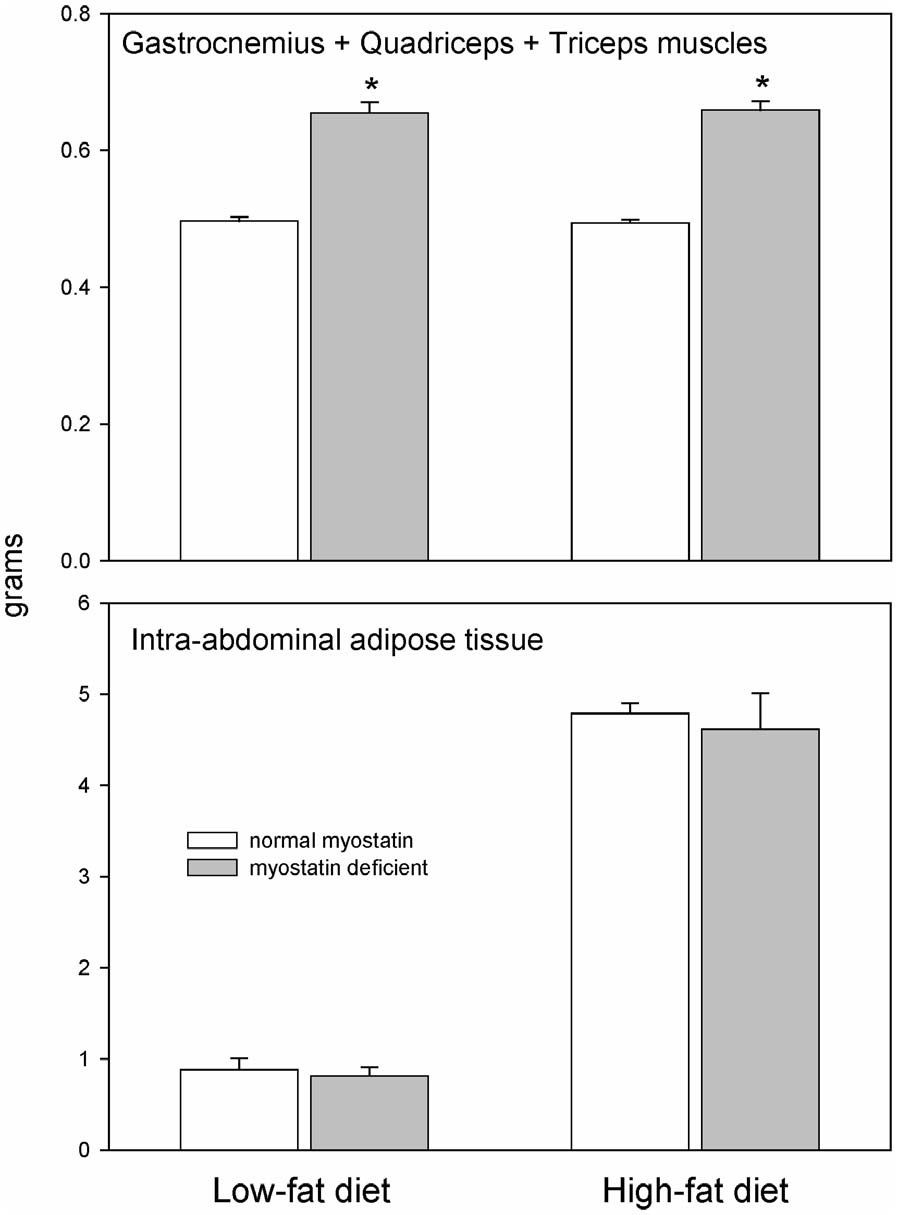
Mean (+SEM) muscle and intra-abdominal adipose tissue mass. Myostatin-deficient (gray bars) and control mice (white bars) were fed a normal low-fat diet (13% of energy from fat) or were fed a high-fat diet (60% of energy from fat) for the final 22 weeks of the experiment. Each bar represents the mean and SEM of 12–15 mice. *P<0.001 versus mice with normal myostatin levels.

At the end of the study, the mass of fat within the abdominal cavity was more than 5-fold greater in the high-fat groups than in low-fat groups (Figure 2). Myostatin depletion did not significantly affect the mass of epididymal or retroperitoneal fat at the end of the study in either the low-fat or the high-fat groups. In mice with normal myostatin genes, the high-fat diet increased hepatic mass by 30% (Table 1). In myostatin-deficient mice, the high-fat diet did not consistently increase hepatic mass (mean+5%, *P* = 0.4). Myocardial mass was increased by the high-fat diet, 14% in control mice and 10% in myostatin-deficient mice. Kidney mass was not significantly affected by the high-fat diet in either normal or myostatin-deficient mice (*P*>0.1, Table 1). We did not weigh the gastrointestinal tract or other organs, but by visual inspection we did not notice any major effects of the high-fat diet or myostatin depletion on the size of internal organs. Skeletal muscle mass (gastrocnemius, quadriceps, and triceps muscles) was about 30% greater than normal in myostatin-deficient mice, as expected, regardless of dietary condition (Table 1, Figure 2).

Of the total difference in mean body mass of 19.9 g between low-fat and high-fat groups with normal myostatin expression (Table 1), less than 5 g can be accounted for by the increased mass of intra-abdominal fat, liver, and heart. Skeletal muscle mass was not increased to any significant extent by the high-fat diet (Figure 2), meaning that we cannot account for ∼15 g of the extra body mass. It was very clear that subcutaneous fat mass was markedly increased after 5 months of high-fat feeding. This compartment might explain most of the unaccounted weight gain, but this was not quantified. The difference in body mass between the low-fat and high-fat myostatin-deficient groups was 14.1 g, and only ∼4 g of this extra weight can be explained by the increased mass of abdominal fat, liver, and heart. Thus, the unexplained portion of the weight difference between myostatin-deficient mice fed the high-fat diet versus those fed the low-fat diet was ∼10 g.

**Figure 3:**
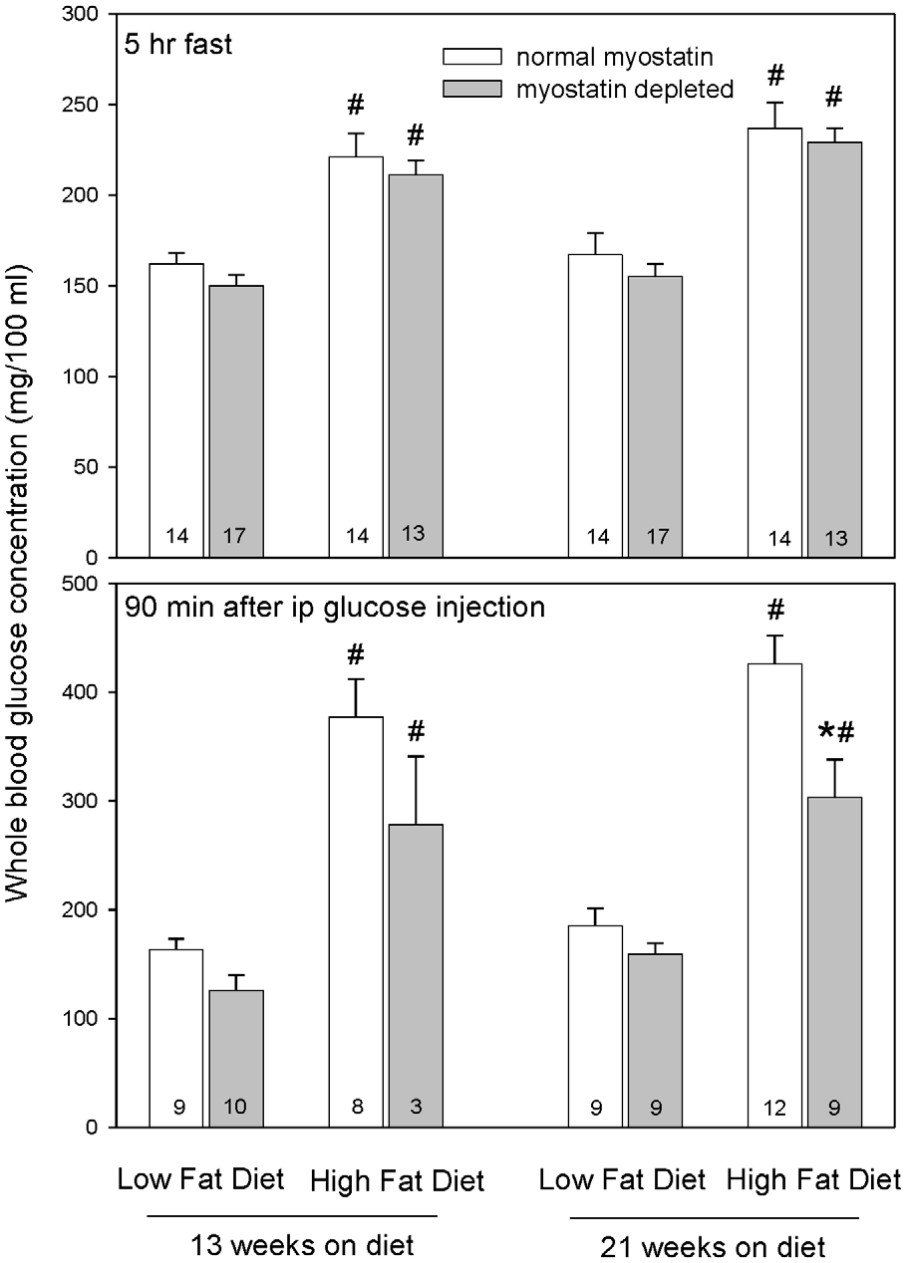
Mean (+SEM) blood glucose concentrations. Bars represent means, whiskers represent SEM. Number of values included in each mean are shown at the bottom of each bar. *P<0.01 vs. normal myostatin group represented by adjacent bar. #*P*<0.02 vs. low-fat group with same myostatin status.

Myostatin depletion did not significantly affect blood glucose levels in mice fed a low-fat diet (Figure 3). As expected, the high-fat diet induced hyperglycemia, both 5 hr after food was withdrawn and 90 min after ip glucose injection (*P*<0.001, Figure 3). In mice fed the high-fat diet for 5 months, myostatin depletion did not affect fasting glycemia but reduced glucose levels 90 min after ip glucose (P<0.01). The same pattern of mean glucose levels was observed after 3 months of high-fat feeding, but 90 min data were available for only 3 myostatin-deficient mice and therefore there was limited power to assess statistical significance.

**Figure 4:**
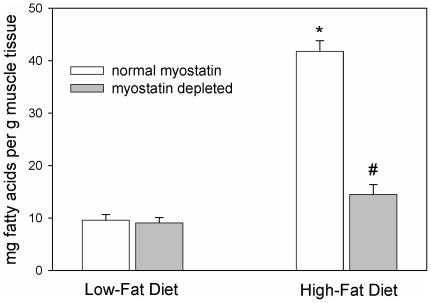
Mean (+SEM) lipid mass in triceps brachii muscles. Esterified + non-esterified fatty acid content (palmitic + palmitoleic + stearic + oleic + linoleic acids, which accounted for ∼90% of total fatty acid mass) was determined by gas chromatography. *P<0.001 vs. low-fat group with normal myostatin expression. #*P*<0.001 vs. high-fat group with normal myostatin expression and *P*<0.05 vs. myostatin-deficient low-fat group. Data from 6 mice per genotype/diet condition.

**Figure 5:**
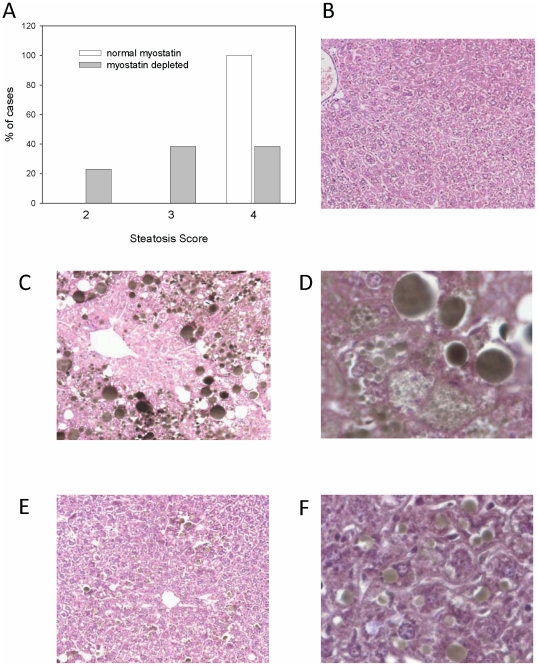
Hepatic steatosis scores and representative micrographs of liver sections. Distribution of steatosis scores (A) is based on examination of 12 mice with normal myostatin expression and 13 myostatin-deficient mice, all of which received the high-fat diet. Mice fed a low-fat diet did not have hepatic fat accumulation (B, Osmium H&E×250). Mice with normal myostatin expression had significant hepatic steatosis after 5 months of high-fat feeding (C×250; D×500). Larger lipid droplets often lift off the tissue leaving the clear spaces seen in the micrographs. Less fat accumulation was evident in livers of myostatin-deficient mice fed a high-fat diet for 5 months (E×250; F×500).

The high-fat diet induced an increase of ∼4-fold in the amount of fat in triceps muscles in mice with normal myostatin levels (Figure 4). In contrast, the high-fat diet increased muscle fat content only 60% in myostatin-deficient mice. The method used to assess muscle fat content did not differentiate between fat within the muscle fibers and fat that accumulated in adipocytes between muscle fibers. Fat deposition in the liver also was attenuated in myostatin-deficient mice (Figure 5). All 12 livers from control mice fed the high-fat diet received the highest steatosis score, whereas only 5 of 13 livers from myostatin-deficient mice received the highest score. The difference between control and myostatin-deficient mice in the distribution of steatosis scores was significant by Fisher's exact probability test (*P*<0.01).

## Discussion

Mice made hypermuscular by postdevelopmental myostatin depletion gained less weight than normal mice during the first few weeks of exposure to a very-high-fat diet. However, the further increase in weight beyond the first few weeks of high-fat feeding was not significantly affected by myostatin depletion. After 5 months of high-fat feeding, the intra-abdominal fat mass was similar in normal and myostatin-deficient mice. The mean total weight gain associated with high-fat feeding (i.e., the difference in final body weights between mice fed high-fat and low-fat diets) was 29% less in myostatin-deficient mice, but we could not account for this difference based on the fat depots and organs that were weighed in this study. Subcutaneous adipose tissue has a very large capacity to store fat and a difference in subcutaneous fat accumulation is a potential explanation for the differential weight gain. Unfortunately, we did not include measurements of subcutaneous fat mass when designing the study because previous research indicated that myostatin knockout or inhibition reduced epididymal, retroperitoneal, and subcutaneous fat mass to the same extent [1], [6], [9], and because of the consensus that central obesity is a more important determinant of metabolic problems than subcutaneous obesity. Future research on the potential anti-obesity effect of inhibiting myostatin activity should evaluate the possibility that subcutaneous adipose tissue is affected more than intra-abdominal adipose tissue after prolonged high-fat feeding.

Previous research has demonstrated that inhibition or deficiency of myostatin does not reduce, and may increase, intake of normal or high-fat chow [5], [6], [9], [14], [20]. Thus it is more likely that increased energy expenditure rather than lower energy intake explains the initial smaller weight gain in myostatin-deficient mice during high-fat feeding. The increased muscle mass induced by myostatin depletion must have increased maintenance energy requirements to some extent. Mature mice with constitutive myostatin knockout had an increase in total oxygen consumption (an index of energy expenditure) of 14% [6]. Recently it was reported that energy expenditure per mouse was increased 33% after 6 weeks of anti-myostatin antibody administration to *ob/ob* mice, in which the increment in muscle mass caused by myostatin blockade was very similar to the increment in muscle mass induced by myostatin depletion in the present study [14].

In mice with constitutive expression of a protein that inhibits myostatin activity, a high-fat diet fed during development (4–23 weeks of age) increased body mass and muscle mass more in myostatin-deficient than in mice with normal myostatin activity [5]. In the present study, the high-fat diet did not enhance the muscle hypertrophy and total weight gain induced by myostatin depletion. Thus, the effect of high energy intake on muscle growth and the influence of myostatin on pathways that regulate this process might be different in immature and mature mice.

Presumably, most of the increase in liver mass associated with high-fat feeding was caused by steatosis, which was evident in the histological examinations of liver sections. The mean increase in liver mass in the obese myostatin-deficient mice (<0.1 g) was markedly attenuated compared with that of the obese mice with normal myostatin expression (0.5 g), and histology indicated that steatosis was not as severe in the myostatin-deficient mice. A constitutive myostatin gene mutation also attenuated the hepatic steatosis induced by a high-fat diet [4]. Blocking myostatin in mice with a soluble activin receptor type IIB reduced hepatic steatosis induced by androgen deprivation [21]. The fact that hypermuscularity induced by elevated Akt1 activity reduced hepatic steatosis in mice fed a high-fat diet [22] suggests that this is an indirect consequence of hypermuscularity rather than a direct effect of reduced activin receptor activity in liver. Myostatin knockout also reduced lipid accumulation in skeletal muscle, an effect that has not been reported previously. The mechanism for reduced fat accumulation in liver and muscles of myostatin-deficient mice is not known and was not examined in the present study.

After a period of fasting, when the serum insulin level is low, muscle consumes fat rather than glucose as its primary fuel source. Thus, it is not surprising that the hypermuscularity in myostatin-deficient mice did not reduce glucose levels after a 5 hr fast. When insulin levels are elevated, muscle becomes a major glucose consumer. The lower glucose levels in myostatin-deficient mice 90 min after glucose administration could simply reflect the increased mass of an insulin sensitive tissue. It also is possible that loss of myostatin increased sensitivity to insulin. Although insulin resistance has been induced by injecting myostatin into mice [4], [23], this does not prove that the normal basal level of myostatin is a significant determinant of insulin sensitivity. Insulin sensitivity typically is defined as the rate of fall of glucose levels after insulin administration, or the amount of glucose that must be infused to maintain constant glucose levels during continuous insulin infusion. Thus, even if the rate of glucose uptake per gram of muscle is not affected by myostatin deficiency, the whole-body insulin sensitivity would appear to be increased because of the larger muscle mass. When the activity of myostatin and other activin receptor type IIB ligands was reduced by systemic administration of a soluble activin receptor type IIB for 10 weeks, there was an increase in insulin-stimulated glucose uptake per g muscle when mice were on a normal diet, but not when they were on a high-fat diet [9]. Nevertheless, the treated mice had lower blood glucose levels on the high-fat diet, perhaps because their leaner body composition improved hepatic insulin sensitivity and thereby reduced endogenous glucose production. Obese *ob/ob* mice treated with an anti-myostatin antibody had reduced glucose levels when consuming normal low-fat food, but this occurred without a change in the glucose-lowering effect of exogenous insulin [14]. More research is needed to clarify to mechanism whereby loss of myostatin activity reduces glucose levels.

All research in this area, including the present study, has examined whether lack of myostatin activity attenuates the development of obesity or hyperglycemia when myostatin activity was knocked out or inhibited before the mice became obese. Future studies should be designed to determine whether inhibition of myostatin signaling can help to reverse established obesity, hepatic steatosis and glucose intolerance.
